# Current situation of neuropathology in Central America

**DOI:** 10.3389/fonc.2024.1378397

**Published:** 2024-10-15

**Authors:** Mónica Núñez Delgado

**Affiliations:** Pathology Department, Hospital Nacional de Niños Dr. Carlos Saénz Herrera, San José, Costa Rica

**Keywords:** neuropathology, brain tumors, pediatric, Central America, immunohistochemistry

## Abstract

The present situation of neuropathology practice in the Central American region has not been addressed in the past. These are low middle-income countries, and therefore, many do not have a basic immunohistochemistry panel. Cytogenetics and molecular studies are not available in most of Central America. Pediatric brain tumors are diagnosed either by anatomical pathologists or by pediatric pathologists. Access to a weakly Latin American Tumor Board is available to consult cases, but most countries do not participate in these expert meetings. The most recent World Health Organization brain tumor book has a very broad molecular classification of pediatric brain tumors. All these factors make it very difficult to properly diagnose pediatric brain tumors in the region, and this impacts the treatment and overall survival of children with brain tumors.

## 1 Background

As the lead pediatric pathologist who has been involved in the diagnosis of pediatric brain tumors for Costa Rica for over 15 years, I have found the evolution of the molecular classification of these tumors to have become overwhelming in a system which does not have all the proper diagnostic tools. The WHO classification of central nervous system tumors has changed three times since I first started practicing. The most recent 2021 WHO fascicle has increased its molecular pediatric brain tumor classification compared with 2016. This has caused a more integrated categorization of pediatric brain tumors, which in previous WHO editions were mostly described along with adult central nervous system tumors. This new classification is important because it considers the great variety of tumors in the pediatric population and how these are unique morphologically as well as from an immunohistochemical and molecular perspective. This is worrisome because in our region, most pathologists are still making diagnosis based on histological patterns alone, which is no longer admissible. All these advances in molecular classification and the lack of proper immunohistochemistry and molecular tools make pathologists feel uncomfortable making a diagnosis of brain tumors in children. Although there is access to weekly meetings with experts from Canada, United States, and Spain through the Latin American Tumor Board where cases are presented and recommendations are given to the treating oncologist, there is a very low participation of pathologists in these meetings. Moreover, although this group also receives a selected number of pathology samples for second review, this is not enough to address the needs of the region.

At Costa Rica’s National Children’s Hospital Dr. Carlos Sáenz Herrera, a broad immunohistochemical panel is available but a series of essential molecular studies are still required. For example, for the classification of gliomas and glioneuronal and neuronal tumors, BRAF V600E is available, but we still lack molecular tools such as BRAF fusions, fusions between MYB or MYBL1 and a partner gene necessary for the diagnosis of MYB- or MYBL1 altered diffuse astrocytoma, as well as deletions and amplifications at the MYB locus on 6q23.3 for the diagnosis of angiocentric glioma and MAPK pathway-activating abnormalities needed in the diagnosis of both polymorphous low-grade neuroepithelial tumor of the young and diffuse low-grade glioma, MAPK pathway-altered (1).

For the proper diagnosis of pediatric-type diffuse high-grade gliomas, such as diffuse midline glioma, diffuse hemispheric glioma, and infant-type hemispheric glioma, H3 K27 and H3 G34 mutations and RTK fusions (NTRK, ROS1, and MET) are required, respectively. DNA methylation profiling is not available and is the only method for establishing a diagnosis of high-grade astrocytoma with piloid features and other tumors such as diffuse glioneuronal tumor with oligodendroglioma-like features and nuclear clusters, as well as rosette-forming glioneuronal tumor, and for the molecular subgrouping of pineoblastoma (1).

We do not have MN1 alteration for a proper diagnosis of astroblastoma, PRKCA fusion needed for papillary glioneuronal tumor, or dinucleotide mutation in the PDGFRA gene required to make the diagnosis of myxoid glioneuronal tumor (1).

For the proper categorization of ependymal tumors, we still require ZFTA and YAP fusions, and for the adequate classification of medulloblastoma, although we have N-MYC and C-MYC (by FISH), we are still lacking DNA methylation analysis, as well as immunohistochemistry for YAP1 and GAB1 (1).

To date, no study or publication has been carried out on the current situation of pediatric neuropathology in developing countries such as Central American countries. It is worrisome because in these countries as well as in the rest of the world, brain tumors in the pediatric age are the most common solid tumors and continue to cause high morbidity and mortality.

It is important to consider that in Central America, most of the population can only access public medicine for economic reasons, so we will only refer to this and not to private medicine where other studies may be available.

Most pediatric brain tumors are diagnosed by general pathologists and pediatric pathologists. In some cases, consultations with adult neuropathologists are carried out because there are no formally trained pediatric neuropathologists in the region.

I carried out a short survey to other participating Central American hospitals who participate in AHOPCA (Asociación Hemato-Oncológica Pediátrica de Centro América) to get a sense of their resources. The main findings where that 40% of the countries do not have immunohistochemistry in general in their Pathology Departments or do not have basic immunohistochemical markers used in the diagnosis of brain tumors and base their diagnosis on morphology alone. The countries that have immunohistochemistry available are Panamá, Costa Rica, Guatemala, and San Salvador. Moreover, for example, although Guatemala has access to some immunohistochemical stains, these are not used for the diagnosis of pediatric brain tumors. The markers available include S100, GFAP, ATRX, olig-2, EMA, enolase, neurofilament, IDH1, p53, and IN1-1, but most countries with immunohistochemistry are still lacking stains such as GAB-1 necessary for proper classification of medulloblastoma and YAP-1 useful in both medulloblastomas and ependymomas, ZFTA which is used in the diagnosis of supratentorial ependymomas, and H3 K27 which is important in diffuse midline gliomas.

More than 85% of the countries do not have access to special tools such as cytogenetics and molecular studies. Those that are available are of very limited use in the diagnosis of brain tumors. For example, Costa Rica has studies such as IDH1, IDH2, 1p/19q codeletion, PTEN, and EGFR which are more useful in the diagnosis of adult brain tumors. N-MYC and C-MYC (FISH) are also available and helpful in embryonal brain tumors in the pediatric population. N-MYC has been available for over a decade because of its implications in the prognosis of neuroblastoma.

It is important to mention that no other publications were found addressing the current situation of neuropathology in Central America. This is the first scientific paper that seeks to analyze the reality this region faces.

In Costa Rica, between 2000 and 2014, the incidence of childhood cancer in children under 15 years of age was 2,396 cases; of these, 13.9% were malignant tumors of the central nervous system, which represents 19.4/million. The highest incidence rates are in children aged 1–4 (22.2/million) and 5–9 years (22.0/million). The incidence of malignant CNS tumors in infants varied between the regions from no cases to 20.8/million. Lower malignant CNS tumor incidence rates were found for most solid tumors, including malignant CNS tumors (4). For medulloblastoma, between the years 2009 and 2015, a total of 31 cases were diagnosed with a 5-year OS rate of 61.3% (3).

It is very challenging to gather outcome data without the existence of pediatric cancer registry in some countries of the region. Most countries in Central America are low middle-income countries and therefore cannot afford to assign staff to keep record of cancer data. Without this information, it is difficult to estimate survival data and mortality in the region (2).

## 2 Conclusions

In summary, there is still a lot that can be done for this region. One option is establishing an outreach program between a specialized center and Central American countries specifically focused on pathology review. Pediatric brain tumors of this region could be presented in a weekly brain tumor board and specific cases, in which the pathology report is unclear, there is a clinical pathologic discrepancy, or if the case requires more immunohistochemical stains or molecular studies for a proper diagnosis, it could be sent out for a second review. This can benefit the patient’s treatment and outcome. Also, it is important for pathologists in this region to have access to proper training in pediatric neuropathology.

I also envision one highly specialized neuropathology center for the diagnosis of pediatric brain tumors located in one country in Central America with at least two trained neuropathologists, and centralization on immunohistochemistry and molecular studies would be more feasible because these are developing countries and the resources and infrastructure required to have the highly specialized equipment and expertise are not feasible to have in most centers. With a project of this nature, all pediatric brain tumor blocks could be sent to one center which would specialize in the diagnosis of pediatric brain tumor of the region. This would be of great benefit for the patients because biopsy results would be more accurate and prompter. Also, specific cases could still be sent out for a second pathology review and to perform specific molecular studies that are only available in highly specialized centers.

Costa Rica has a socialized healthcare system, and the government invests in this health system. This has made it possible for us to have access to more diagnostic tools. It has been a long journey finding providers that are willing to bring the immunohistochemical stains necessary for the diagnosis of pediatric brain tumors because these are not so widely, and the economic cost is high. Countries in Central America could partner with developed countries in the diagnosis of brain tumors and invest in one large center in the region where the expertise, a broad panel of immunohistochemical markers, and molecular studies would be available.

Central America is a region that definitively would benefit from an outreach program with a highly specialized center in the United States, Europe, or Canada, and this would be of high impact in making treatment decisions and in the overall outcome of children with brain tumors in the region.

## Data Availability

The original contributions presented in the study are included in the article/supplementary material. Further inquiries can be directed to the corresponding authors.

## References

[1] Louis DN Perry A Wesseling P Brat DJ Cree IA Figarella-Branger D. The 2021 WHO classification of tumors of the central nervous system: a summary. Neuro-oncology. (2021) 23:1231–51. doi: 10.1093/neuonc/noab106 PMC8328013 34185076

[2] Díaz-Coronado R Villar RC Cappellano AM. Pediatric neuro-oncology in Latin America and the Caribbean: a gap to be filled. Front Oncol. (2024) 14:1354826. doi: 10.3389/fonc.2024.1354826 38571497 PMC10989070

[3] Sánchez S Gamboa Y Rodríguez C. Caracterización clínica y epidemiológica de los pacientes pediátricos con diagnóstico de meduloblastoma tratados en el Servicio de Oncología del Hospital Nacional de Niños desde enero de 2009 hasta diciembre de 2015. Universidad de Costa Rica. SIBDI (2017).

[4] Erdmann F Li T Luta G Giddings BM Torres Alvarado G Steliarova-Foucher E. Incidence of childhood cancer in Costa Rica, 2000-2014: An international perspective. Cancer Epidemiol. (2018) 56:21–30. doi: 10.1016/j.canep.2018.07.004 30025251

